# Optimal social distancing in epidemic control: cost prioritization, adherence and insights into preparedness principles

**DOI:** 10.1038/s41598-024-54955-4

**Published:** 2024-02-22

**Authors:** Giulio Pisaneschi, Matteo Tarani, Giovanni Di Donato, Alberto Landi, Marco Laurino, Piero Manfredi

**Affiliations:** 1 Department of Information Engineering, University of Pisa, Pisa, Italy; 2 Institute of Clinical Physiology, National Research Council, Pisa, Italy; 3 Department of Economics and Management, University of Pisa, Pisa, Italy

**Keywords:** Viral infection, Ecological epidemiology

## Abstract

The COVID-19 pandemic experience has highlighted the importance of developing general control principles to inform future pandemic preparedness based on the tension between the different control options, ranging from elimination to mitigation, and related costs. Similarly, during the COVID-19 pandemic, social distancing has been confirmed to be the critical response tool until vaccines become available. Open-loop optimal control of a transmission model for COVID-19 in one of its most aggressive outbreaks is used to identify the best social distancing policies aimed at balancing the direct epidemiological costs of a threatening epidemic with its indirect (i.e., societal level) costs arising from enduring control measures. In particular, we analyse how optimal social distancing varies according to three key policy factors, namely, the degree of prioritization of indirect costs, the adherence to control measures, and the timeliness of intervention. As the prioritization of indirect costs increases, (i) the corresponding optimal distancing policy suddenly switches from elimination to suppression and, finally, to mitigation; (ii) the “effective” mitigation region—where hospitals’ overwhelming is prevented—is dramatically narrow and shows multiple control waves; and (iii) a delicate balance emerges, whereby low adherence and lack of timeliness inevitably force ineffective mitigation as the only accessible policy option. The present results show the importance of open-loop optimal control, which is traditionally absent in public health preparedness, for studying the suppression–mitigation trade-off and supplying robust preparedness guidelines.

## Introduction

The response to the COVID-19 pandemic, described as “a massive global failure at multiple scales” by the Lancet Commission on Future Preparedness^1^, has highlighted the vulnerability of modern public health systems to pandemic events. Notably, although the COVID-19 pandemic was more dramatic than expected in previous pandemic preparedness plans—mostly focused on influenza^2^—it cannot be taken to be ’the worst of the worst‘. This scenario could be represented by a highly transmissible pathogen with (i) short generation and doubling times (as was the case for COVID-19), (ii) high mortality rates in the young and adults and (iii) ability to mutate rapidly. However, complicated control scenarios might also occur for less transmissible pathogens whose risk is perceived as low in large segments of the population, causing low adherence in wide population groups. Facing such diverse scenarios will require a jump in preparedness science to fully embrace new dimensions such as holism and sustainability in societal protection and solidarity within and between countries^1^.

In relation to future preparedness, the COVID-19 experience highlighted the tension between the duration of control measures and societal feedback, as was already apparent during the generalized lockdowns adopted to control the first wave of the epidemic in early 2020. This has increased the need to better understand the trade-off between the direct health impact of epidemics (e.g., hospitalizations, deaths and overwhelming public health resources) and their indirect effects, i.e., the societal, economic, health and relational damage resulting from control measures^3,4^.

Regarding the measures used for balancing this trade-off, different control options have emerged since the first COVID-19 wave. Early seminal work mainly distinguished between mitigation and suppression^5^. The former strategy aims to delay (but not necessarily reverse) the spread of the epidemic to reduce pressure on the healthcare system while protecting the most vulnerable ones and eventually generating herd immunity. Instead, the latter aims to reverse epidemic spread and reduce case numbers to low levels without stopping community transmission. In this case, measure relaxation implies a case rebound calling for a boosting of restrictions, in the form of intermittent control. However, a number of studies have proven that elimination i.e., ending community transmission, is feasible^6,7^ through appropriate actions and is economically rewarding^7^. During the COVID-19 pandemic, governments’ positions on the issue were far from clear-cut, ranging from elimination, adopted by China, New Zealand and Australia, to suppression strategies adopted by many European countries, to the mitigation option initially invoked by the UK and US governments to pursue herd immunity and maintained for a long time by Sweden^8^. Even now, there does not seem to be full consensus about the optimal action.

Relatedly, we agree with the underrated observation that despite the large amount of epidemic modelling efforts available already before the COVID-19 pandemic, we still lack clear principles for robustly comparing different control strategies from a preparedness viewpoint^9^. This is of paramount importance given the difficulties in implementing optimal real-time modelling/intervention under the emergency/urgency conditions that prevail once an outbreak of an unknown pathogen is ongoing. In relation to this, open-loop optimal control represents an ideal tool for setting up the best preparedness baseline plans for future epidemics^9,10^. After describing the problem via a mathematical model of the epidemic, which includes possible policy actions over a certain control horizon, one sets an appropriate cost function combining both the direct costs of the epidemic and those of the control actions and seeks the optimal time trajectories that minimize costs. In the case of preparedness, the control action must be open-loop given that no direct measures are available on the actual epidemic course. Nevertheless, the control parameters can be varied to create different scenarios and predict the best actions to take.

Pre-COVID-19 applications involving optimal control of communicable infections have typically considered^11–16^ specific interventions (e.g., vaccination) against a given infection as an isolated process within an otherwise unaffected community. This reductionist approach has the drawback that it disregards critical societal phenomena, such as public health resource saturation. There have been few exceptions to this^17,18^. Reductionism reflects a widespread attitude in the public health systems of modern industrialized countries, largely due to the nonthreatening nature of communicable diseases in such settings^19^. The devastating impact of COVID-19 has revived optimal control studies, e.g., by considering the optimal allocation of multiple interventions, their prioritization, the protection of finite public health resources (e.g., hospitals) and ultimately addressing the aforementioned trade-off between direct and indirect costs. In this regard, an impetus was provided by economists who first included overall economic loss due to generalized lockdowns^20,21^.

With a special focus on control actions, the COVID-19 experience has further confirmed that until effective vaccines become available, social distancing remains the key control measure when the epidemic proves uncontrollable, leaving a secondary role to other interventions^3,5,22–24^. Numerous contributions have addressed the issue of optimal COVID-19 control through social distancing^9,10,20,21,25–40^. These analyses varied by (i) the type of epidemic model, ranging from simple (e.g., SIR) to detailed ones; (ii) the form of the cost function, from the generic implicit “u(t) cost” to explicit ones, including socioeconomic evaluations (e.g., number of working hours lost due to lockdowns); (iii) the type of trade-off representation, i.e., single- vs multiobjective; and (iv) the type of control problem, i.e., open vs closed-loop.

Given the critical role played by social distancing, we aim to contribute to the emerging debate on future preparedness by thoroughly investigating how the temporal shape of optimal social distancing depends on three main policy factors, namely (i) the *prioritization attributed to indirect epidemic costs*, taken as a free parameter varying between $0$ (full prioritization of direct costs) and $1$ (full prioritization of indirect costs); (ii) the *adherence* of the population to the proposed control measures; and (iii) the *timeliness* with which control actions are enacted following early alerts. Consistent with the aim of seeking “control principles”, we present an exhaustive analysis of the dependence of optimal social distancing on these three key factors.

The problem is formulated as a finite horizon, single objective problem where the cost functional combines the direct epidemiological costs of the epidemic with its indirect costs, inspired by influential economic efforts^20,21^. For transmission, an ordinary differential equation (ODE) model for the first wave of COVID-19 in one of its most aggressive settings, namely, Italy—the second country worldwide shot by the pandemic tsunami—was chosen^41^. The model includes all those features that can make an emerging virus difficult to control^42,43^, namely, presymptomatic and asymptomatic transmission, differential severity, and finite hospital capacity in a context of high transmission and short doubling times. Hospital saturation is addressed by introducing a new class of untreated people suffering higher mortality than hospitalized individuals.

The results provide a detailed characterization of how optimal social distancing trajectories depend on different combinations of cost prioritizations, adherence and timeliness as well as insights into their implications for pandemic control options.

## Results

We report the shape of the optimal social distancing action $L(t)$, representing the fraction of the population targeted for mandatory restrictions at any time during the control horizon $T$ (set to 1 year) that optimally balances direct and indirect costs. We also report the corresponding key epidemiological outputs, such as infection incidence and people needing hospitalization. In particular, we analyse the pattern of optimal distancing $L(t)$ across the entire admissible region for the three aforementioned critical factors, i.e., (i) the prioritization attributed to indirect costs, represented by a single parameter $\Lambda$: for $\Lambda =1$ ($\Lambda =0$), the government fully prioritizes indirect (direct) costs; (ii) the adherence to restrictions ($\theta$): for $\theta =1$, adherence is maximal, i.e., all targeted people adhere to social distancing, while lower values imply lower adherence; and (iii) the timeliness of intervention, i.e., the number of days of free epidemic growth before optimal control is enacted.

To adequately classify and compare optimal social distancing trajectories in terms of possible control options, namely, mitigation, suppression, and elimination^5,44^, we preliminarily need suitable definitions of these concepts from a modelling viewpoint i.e., based on key epidemiological parameters such as reproduction numbers. We recall a few useful definitions: (i) basic reproduction number (BRN, or R_0_), the number of secondary cases caused by a typical infective individual in a fully susceptible population in the absence of intervention measures; (ii) control (basic) reproduction number (CRN, or R_0,C_), the number of secondary cases caused by a typical infective in a wholly susceptible population in the presence of control measures of overall efficacy C; and (iii) effective reproduction number (ERN, R_E_(t) or R_eff_), the number of secondary cases caused by a typical infective at the current levels of susceptibility and adopted measures. Borrowing from recent work investigating the dichotomy suppression-mitigation for different cost combinations^39^, we term mitigation a strategy in which the CRN is never deliberately brought below threshold, epidemic spread is not reversed, and the ERN goes below the unit threshold only by eventual acquisition of immunity, thereby bringing the epidemic to an end. Instead, suppression aims to halt transmission, bringing the CRN (and the ERN) below the threshold. Clearly, as the restrictions involved cannot be prolonged indefinitely due to indirect costs, the epidemic will rebound when measures cease. Both such patterns will endogenously emerge from our analyses. The case of elimination is more subtle because this strategy cannot be investigated by the standard optimal control approach based on an ODE transmission system. Nonetheless, the optimal solution in the relevant parametric region (i.e., at high levels of prioritization on direct costs and adherence) shows—in the initial part of the horizon—a consistent pattern characterized by intense and timely social distancing until incidence is brought to such negligible levels that an appropriate stochastic model would almost always lead to elimination. Therefore, although elimination appears to be a sound consequence of our model within a specific cost range, beyond the aforementioned levels, the predictions of the deterministic model become invalid. In this case, phenomena predicted by the deterministic model, such as long-term epidemic rebounds, are just artefacts. With this warning, we retained the wording “elimination” to identify the corresponding regions in the graphic outputs. Additionally, compared to the cited work^39^, which did not consider public health constraints, the inclusion of a fixed hospital capacity can cause the optimal solution to maintain reproduction at (or very close to) the threshold level for a long interval of time to maintain constant hospital occupancy, as detailed in the Supplementary Materials (SM).

### Effect of prioritization on indirect costs

**Figure 1:**
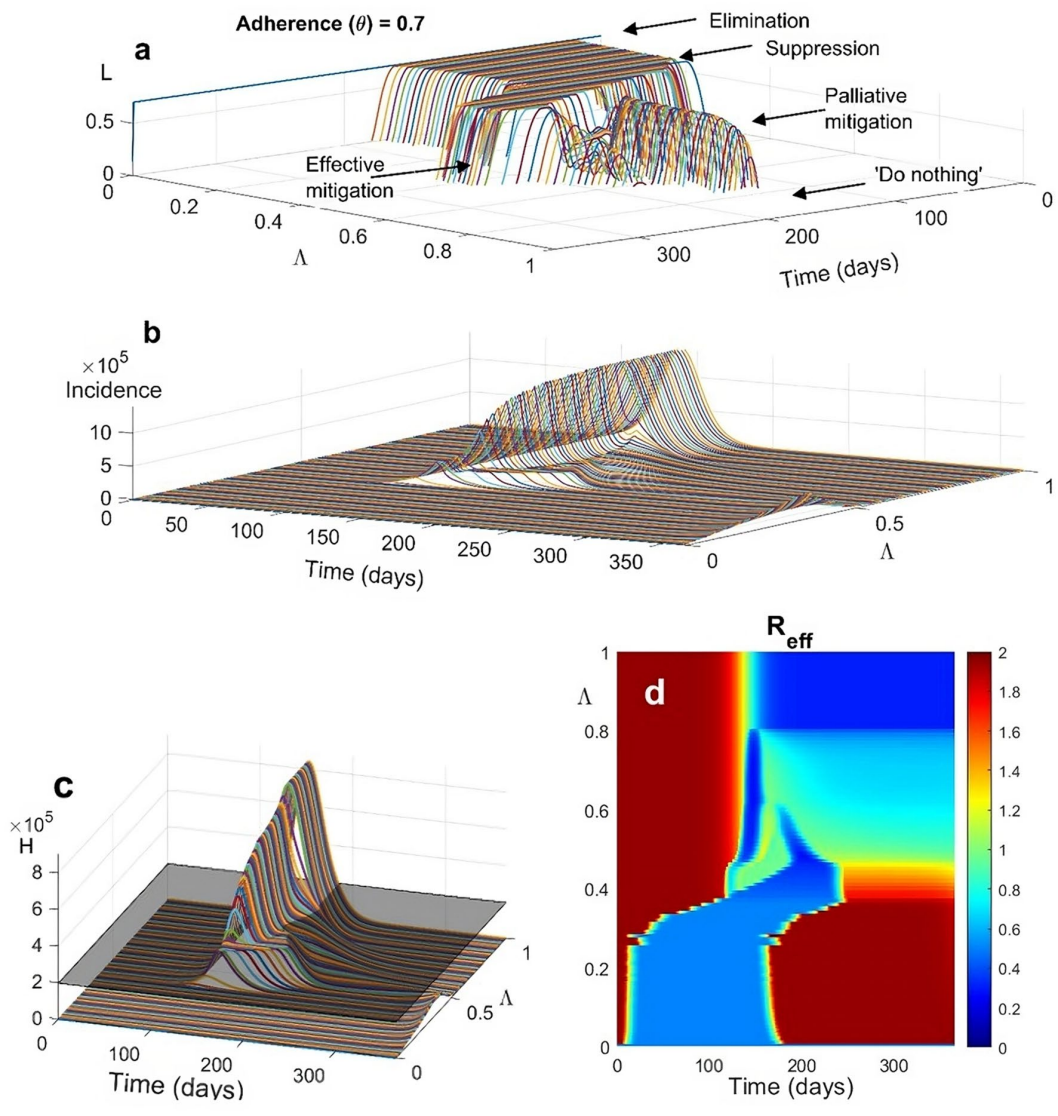
Temporal trends of (**a**) optimal social distancing $L(t)$, (**b**) the corresponding incidence of new infections $\lambda S{\left(1-\theta L\right)}^{2}$, (**c**) people needing hospitalization (compartment H), and (**d**) effective reproduction number for different values of prioritization of indirect costs $\Lambda$. The shaded plane in the H graph represents the maximum hospital capacity. The maximal fraction of people targetable for social distancing is set to ${L}_{max}=0.7$, and the population adherence to social distancing (θ) is set to 70%. All the other parameters and initial conditions are reported in the Methods section. As $\Lambda$ switches from $\Lambda =0$ (full prioritization on direct costs) to $\Lambda =1$ (full prioritization on indirect costs), optimal social distancing undertakes the entire set of switches from elimination to “do nothing” (**a**).

Figure 1 shows the emerging sequence of windows of optimal social distancing for different levels of $\Lambda$ (Fig. 1a) and an adherence of 70% ($\theta =0.7$) under the ideal situation where timeliness is maximal, i.e., the alert system allows early detection of the epidemic and intervention is undelayed:

(i) A *high degree of prioritization of direct costs* $\left(0\le\Lambda \le {\Lambda }_{1},{\Lambda }_{1}\cong 0.37\right)$ leads to elimination according to the above definition. The optimal control $L(t)$ has a timely bang-bang shape i.e., it soon rises to its maximum, stays constant for a while and finally decreases quickly until it vanishes. In all these situations, there is a time when social distancing brings incidence to very low levels, making subsequent predictions invalid. In particular, at full *prioritization of direct costs* $(\Lambda =0)$, the control function $L(t)$ sets to its maximum (${L}_{max}$) for the entire horizon, which is not surprising given that in this case, the trade-off between costs disappears;
(ii) *Intermediate prioritization of direct costs* (${\Lambda }_{1}<\Lambda \le {\Lambda }_{2}, {\Lambda }_{2}\cong 0.46$) yields true *suppression*. Notably, the transition from elimination to suppression occurs almost suddenly in the parameter space, what we call a “razor-blade” effect. The optimal social distancing is gradually delayed for different $\Lambda$ values (compared to case (i)) but maintains a bang-bang shape of almost constant duration. In this window, hospitals’ capacity is never overwhelmed (Fig. 1c). Specifically, there is a subregion $\left({\Lambda }_{1}<\Lambda <0.42\right)$ where suppression is strong, i.e., the epidemic spread is reversed before hospital capacity is saturated. For larger values, suppression becomes weak i.e., there is a period during which hospitals operate at full capacity (although never overwhelmed) because the incidence stays essentially constant (Fig. 1b). The ERN (Fig. 1d), initially reduced by available contact tracing to a level of approximately 2.0, falls below the unit threshold and remains low for a large part of the horizon before eventually rebounding due to lifting measures with increasing indirect costs.
(iii) Beyond $\Lambda ={\Lambda }_{2}$, a transition from *suppression to mitigation* occurs. In particular, there is a narrow window (approximately $0.46<\Lambda \le {\Lambda }_{3}, {\Lambda }_{3}\cong 0.49$), where the epidemic is mitigated through a long-lasting period in which hospitals are saturated but almost never overwhelmed. We term this scenario “*effective mitigation*” because a substantial amount of immunity is created. Surprisingly, in this scenario the optimal action is characterized by a low-intensity (${L\left(t\right)\ll L}_{max}$), short duration, initial wave of intervention followed by a relaxation phase and by a harsher intervention wave at a later stage. This 2-wave shape of the optimal policy is due to the hospitals’ occupancy constraints, and indeed, it does not occur when no constraint is considered (see the SM for details).
(iv) *Increasing prioritization of indirect costs* (approximately ${\Lambda }_{3}\le\Lambda \le {\Lambda }_{4}, {\Lambda }_{4}\cong 0.61)$ substantially delays interventions, making mitigation increasingly ineffective, or *palliative*, causing faster and larger epidemics, overwhelming hospitals, and building-up a wide population of untreated individuals, represented by the portion of the H curve exceeding the capacity boundary (Fig. 1c). In this case, optimal social distancing maintains the 2-wave form, though with a reverse shape: the first wave becomes more intense (to counterbalance the delayed action).
(v) A *large prioritization of indirect costs* yields first to the disappearance of the multiple wave pattern (${\Lambda }_{4}\le\Lambda \le {\Lambda }_{5},\boldsymbol{ }\boldsymbol{ }{\Lambda }_{5}\cong 0.80$), with a dramatic decline in the timeliness and severity of social distancing, and subsequently ($\Lambda >{\Lambda }_{5})$ to the '*do nothing'* policy^5^, with rapid achievement of herd immunity.

Notably, the windows of the patterns reported in Fig. 1 are qualitatively robust to changes in initial conditions and other parameters.

**Figure 2:**
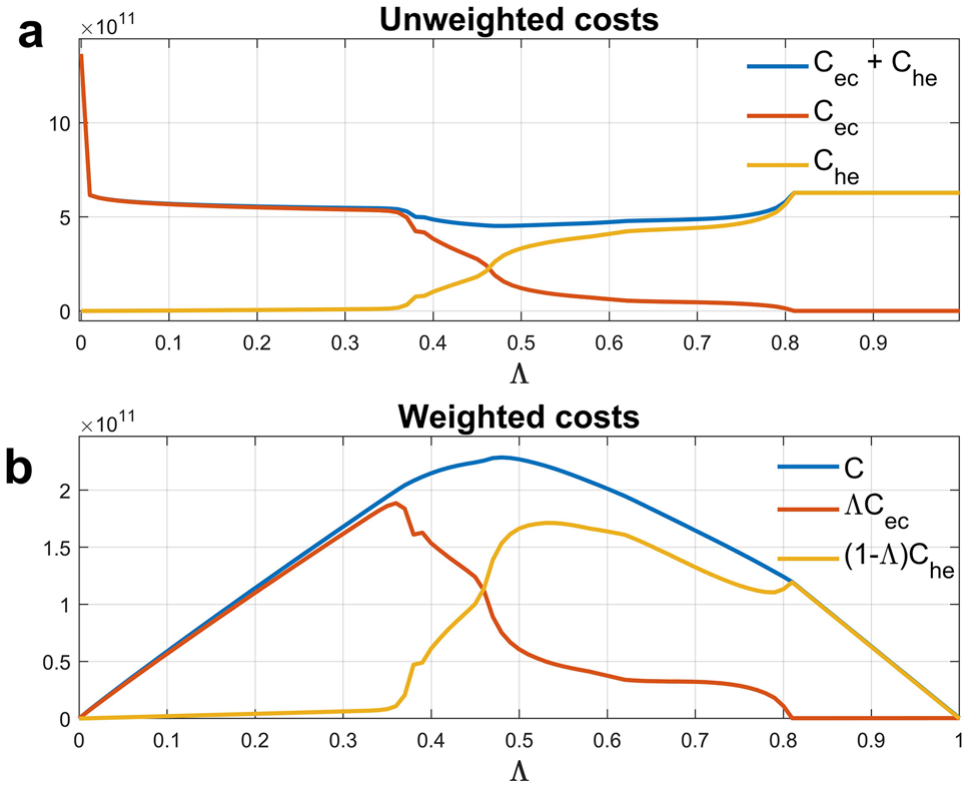
(**a**) true unweighted epidemic costs C_he_ = direct health cost of the epidemic, C_ec_ = indirect (economic) costs, C_he_ + C_ec_ = total (unweighted) costs as functions of parameter $\Lambda$ reflecting prioritization for indirect costs. (**b**) the corresponding weighted costs $(1-\Lambda )$ C_he_, $\Lambda$ C_ec_, C=$(1-\Lambda )$ C_he_+ $\Lambda$ C_ec_, also drawn as functions of $\Lambda$.

The corresponding pattern of the unweighted cost components (i.e., true indirect costs vs. true direct costs vs. their unweighted sum) for different values of $\Lambda$ (Fig. 2a), which reflects the trade-off between the direct and indirect costs of the epidemic net of the decision maker’s preferences, provides an alternative view of previous findings. First, the optimal solution for $\Lambda =0$ i.e., maximum control for the entire horizon yields enormous indirect costs without providing any clear advantage in terms of epidemic control compared to nearby elimination policies. Indeed, the latter allows a sudden, dramatic, drop in indirect costs that decline slowly thereafter due to the strongly similar shapes of the optimal control actions. Furthermore, at the razor blade, marking the rapid transition from elimination to suppression ($\Lambda =0.38$), direct (indirect) costs begin to rise (fall) quickly. The regime changes in the cost components for $\Lambda >0.38$, where direct (indirect) costs initially increase (decline) faster, slow down and then accelerate again, reflect various phase changes observed in Fig. 1 i.e., the switch between suppression and effective mitigation $\Lambda \cong 0.46)$ and subsequently to palliative mitigation ($\Lambda \cong 0.49)$. In the palliative mitigation region, the further discontinuity in the cost speed ($\Lambda =0.61)$ marks the disappearance of the 2-wave regime and the return to single-wave optimal control. Finally, the last acceleration in costs leads to the disappearance of any mitigation intervention i.e., the “do nothing” scenario ($\Lambda \cong 0.80)$. The corresponding weighted components (Fig. 2b), which additionally reflect the weighting by the decision maker's preferences, amplify the aforementioned behaviour.

### Adherence (${\varvec{\theta}}$)

**Figure 3:**
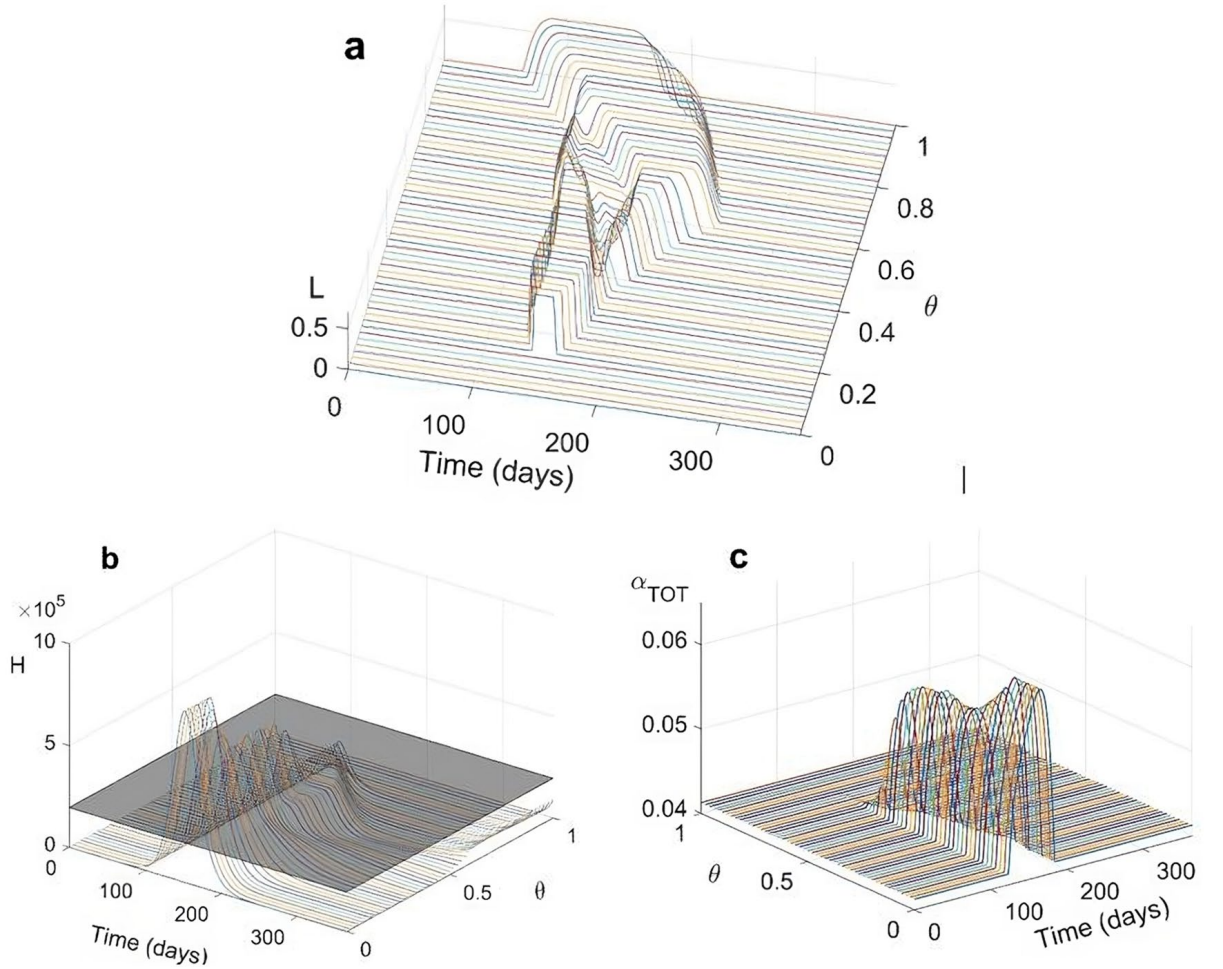
Effects of population adherence to interventions ($\theta$) on optimal social distancing for $\Lambda =0.42$. (**a**) temporal trends of optimal social distancing $L(t)$ as a function of $\theta$; (**b**) corresponding temporal trends of people requiring hospitalization (H(t)) as a function of $\theta$; (**c**) temporal trends of the overall death rate ${\alpha }_{TOT}(t)$ as a function of $\theta$.

Given the level of prioritization on indirect costs $(\Lambda )$, the fraction of the population adhering to the proposed measures $(\theta )$ becomes critical. For example, at intermediate $\Lambda$ levels ($\Lambda =0.42$), the optimal action is always delayed (Fig. 3a). In this case, only very high adherence $(\theta >{\theta }_{1}\cong 0.80$) can promote elimination. Decreasing levels of adherence generate a sequence of scenarios similar to those reported in Fig. 1: (i) for ${\theta }_{2}<\theta <{\theta }_{1}$ (${\theta }_{2}\cong 0.73$) suppression occurs in the *strong* form previously defined while for ${\theta }_{3}<\theta <{\theta }_{2}$ (${\theta }_{3}\cong 0.70$) *weak* suppression appears, again with multiple optimal control waves; (ii) at lower adherences (${\theta }_{4}<\theta <{\theta }_{3}$, ${\theta }_{4}\cong 0.66$), the optimal mitigation action becomes increasingly ineffective, allowing large epidemics and hospitals overwhelming, yielding in turn a blow-up of untreated individuals (Fig. 3b) and a mortality wave of increasing height and duration (Fig. 3c). Further details for alternative $\Lambda$ values (Supplementary Materials) robustly confirm these patterns.

### Joint effects of cost prioritization and adherence

**Figure 4:**
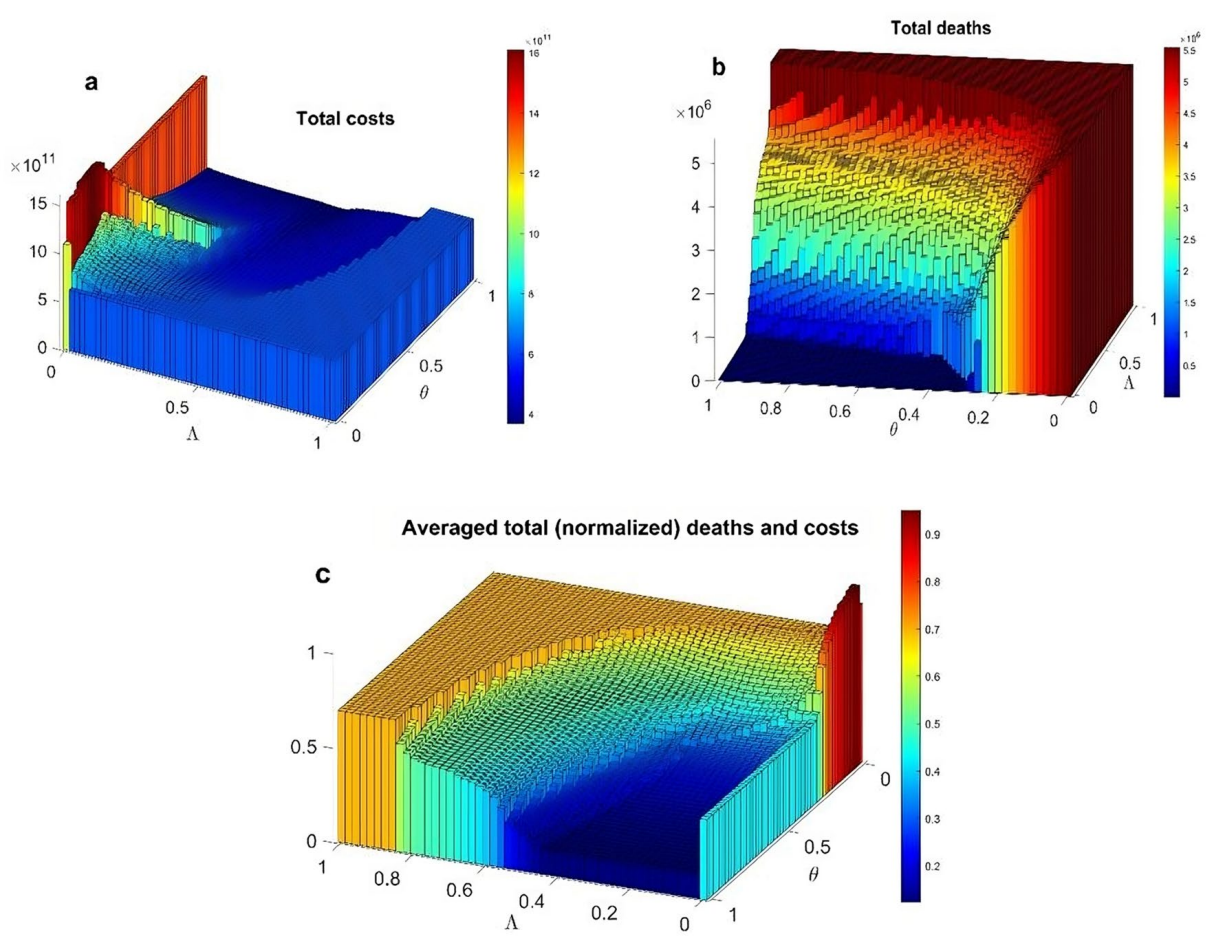
Various aggregated metrics of epidemic costs as functions of prioritization of indirect costs ($\Lambda$) and adherence to the proposed measures $(\theta )$. (**a**) total unweighted costs; (**b**) cumulative COVID-19-related deaths at the end of the horizon D(T); (**c**) average of the two previous normalized metrics. Normalization in the bottom panel is used to avoid the disproportionate influence of either component that arises in some of the parametric regions considered.

A more complete overview of the dependency of optimal social distancing on parameters $\left(\Lambda , \theta \right)$ is reported in Fig. 4, using the cumulative cost and deaths at the end of the horizon as metrics. The flat “low deaths” zone (blue) at low prioritization of indirect costs and high adherence levels corresponds to the region where the optimal action is either elimination or suppression. However, when the context worsens i.e., either adherence or prioritization to indirect costs are lower, the optimal action sets into the region of palliative mitigation and eventually into the “do-nothing” zone. When adherence is low, not even a very strong prioritization of direct costs can make suppression as the optimal solution. Examining the total unweighted costs (Fig. 4a) in the same spirit as Fig. 2a reveals a wide region where elimination/suppression coexists with relatively low total costs. Correspondingly, the average of the two previous metrics (Fig. 4c) shows a wide region (the blue region) where a sufficiently high level of adherence prioritizes health protection, making elimination/suppression the far better societal solution, yielding the lowest number of infection-related deaths and a relatively short duration of restrictions (as shown in Fig. 3a).

### Timeliness: effects of intervention delays

By *timeliness* we denote the ability of public policy to intervene as early as possible during a pandemic emergency^5^. The results presented in the preceding subsections address the ideal scenario of maximal timeliness, wherein there is no alert delay (e.g., due to a lack of knowledge about the initial epidemic course) or an intervention lag by public authorities.

**Figure 5:**
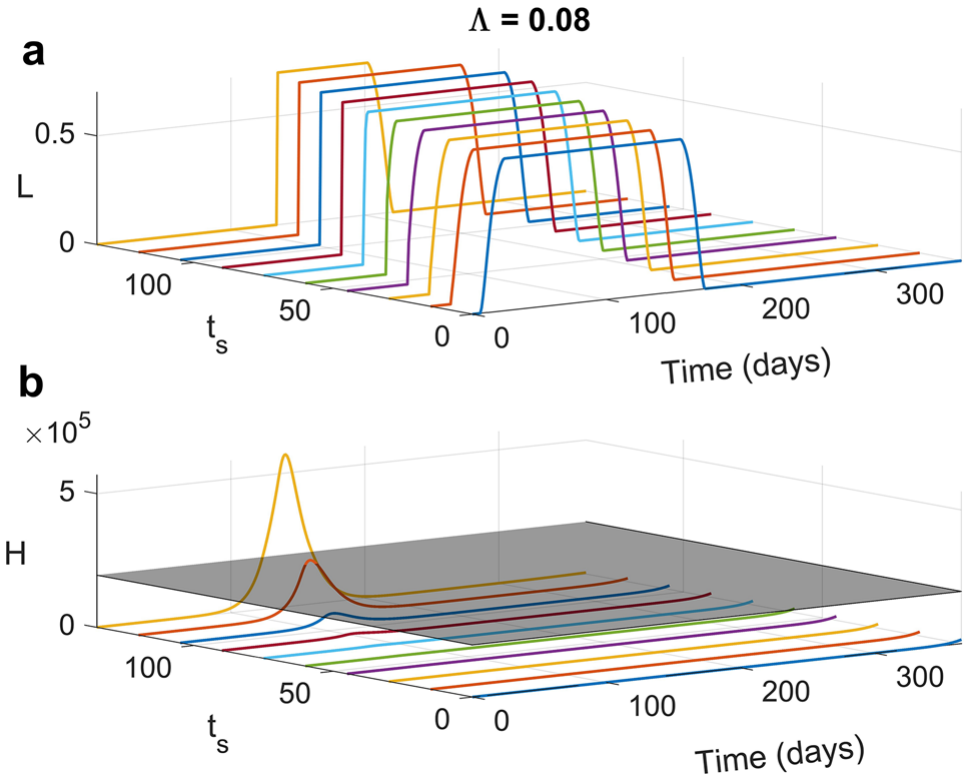
Effects of different intervention delays (${t}_{s}, \,in \,days$) under a regime of strong prioritization of direct costs ($\Lambda =0.08)$ and high adherence ($\uptheta =0.70$). (**a**) temporal trends of optimal social distancing L(t); (**b**) number of people requiring hospitalization H(t).

In real situations, substantial delays are almost inevitable for infections (as was the case for COVID-19) characterized by latent and silent transmission and confirmatory testing. Other things being equal (e.g., in the absence of uncoordinated behavioural responses at the individual level that compensate for the inaction of public policy), intervention delays will result in an initial phase of uncontrolled epidemic growth until a certain time $t={t}_{s}$ when the optimal distancing policy is implemented. The implications are as follows. First, if the underlying parametric configuration ($\Lambda ,\uptheta$) forces the optimal policy in the mitigation region in the undelayed case (i.e., suppression is not an available option), then the optimal control is not affected and remains as such until the end of the horizon. This result is consistent with Bellman’s optimality principle, which states that subsets of optimal actions (and their corresponding trajectories) are also optimal for the underlying subproblem whose initial conditions belong to the optimal path^45^. Therefore, we do not report results for this case. On the other hand, if—in the absence of intervention delays—the underlying parametric constellation ($\Lambda ,\uptheta$) sets the optimal policy in the suppression (or elimination) region, the problem must be reset. Figure 5 reports the optimal social distancing (a) and the corresponding trajectory of people requiring hospitalization H (b), for different values of the intervention delay ${t}_{s}$ under a combination of a high prioritization of direct costs $\left(\Lambda =0.08\right)$ and large adherence ($\uptheta =0.70$). In the absence of intervention delays, such values promote elimination (Fig. 1). For nonlarge delays ${t}_{s}$ (${t}_{s}\le 70\, days)$, the optimal distancing schedule is temporally shifted compared to the unlagged case, and either elimination is maintained or modified into suppression. However, for longer delays (${t}_{s}>70 \,days$), a rapid transition from suppression to palliative mitigation occurs (Fig. 5b). In other words, due to the narrowness of the window of effective mitigation, the temporal range of intervention delays compatible with effective mitigation is also dramatically narrow, meaning that large delays leave ineffective mitigation as the only available policy option.

## Discussion

Pandemic preparedness plans developed worldwide since the early 2000s, were based primarily on data from the 1918 Spanish flu and secondarily from the mild 2009 H1N1 pandemic. However, the first wave of COVID-19, exhibited greater transmissibility and shorter doubling times than did the Spanish flu virus^2^. Additionally, no logical argument can rule out the possibility that a future pandemic could even be more severe than COVID-19. Furthermore, even less transmissible pathogens can be difficult to control if adherence to measures is low, for example, when risks are perceived as low in large segments of the population. Therefore, improving our understanding of the most effective interventions for balancing the direct effects of a threatening outbreak with its indirect effects, i.e., the disruption of social, economic and relational ties, is a critical priority for future preparedness.

Based on the observation^9^ that the epidemiological community still lacks generally agreed upon principles for comparing different intervention options from a preparedness standpoint, this article used open-loop optimal control to contribute to the general understanding of the forms and implications of optimal social distancing. After the onset of the COVID-19 pandemic, optimal control techniques have been extensively used to assess the effectiveness of different types of undertaken measures, particularly social distancing, accounting for several economic or societal considerations^9,20,21,25–40^. This work aims to contribute to this vein by especially focusing on social distancing actions that optimally balance the trade-off between direct and indirect costs, in the spirit of developing principles useful for future preparedness.

In particular, we searched for the optimal social distancing trajectories that minimize a generic combination of direct and indirect costs and assessed the dependence of the optimal action on three key policy factors, namely, (i) the degree of prioritization of indirect costs, (ii) population adherence to control measures, and (iii) the timeliness of intervention. This makes our objectives complementary to a recent contribution also focusing on cost prioritization^39^, which instead analyses the effects of transmission, including how optimal social distancing changes for less transmissible pathogens, and horizon length though in a simple SIR model without public health constraints and adherence.

Our main results provide insight into the interplay between cost considerations and intervention parameters in shaping optimal social distancing and the suppression-mitigation trade-off. First, when the prioritization of direct costs is very high and adherence is adequate, the optimal action is harsh and can be naively interpreted as elimination. Indeed, after the incidence has been brought to very low levels, the deterministic model is no longer adequate. Second, as the prioritization of indirect costs increases, the corresponding optimal action shows a rapid phase transition from elimination to suppression. Such rapid transitions have also been identified in recent research^39^. Third, as the prioritization of indirect costs further increases, the optimal action shows a rapid “phase” transition from suppression to mitigation. Notably, the parametric region in which mitigation is effective (i.e., preventing hospitals’ overwhelming) is very narrow even at the hospital size scale in Western countries. After this region of effective mitigation, further increasing prioritization of indirect costs makes mitigation ineffective, with increasing numbers of people that cannot be cured due to hospital burdens. This is a crucial element for determining which strategy should be implemented. Additionally, in the “effective mitigation” region, the optimal social distancing policy shows multiple waves. This results from the attempt to balance direct and indirect costs under the finiteness of public health resources in the presence of epidemic inertia. In particular, the second wave of restrictions is harsher than the first wave. This result is interesting in terms of the public communication required to maintain high adherence in a population just exiting from a first wave of restrictions. Finally, we highlight the delicate balance between prioritizing indirect costs and both adherence and timeliness: given the narrowness of the effective mitigation region, high prioritization of indirect costs and low adherence unavoidably tend to leave ineffective mitigation as the only option. This is in turn worsened by intervention delays.

As an additional remark, unlike adherence and timeliness which have direct control meanings, the prioritization of indirect costs ($\Lambda$) is more subtle. Indeed, during an actual epidemic, the prioritization of one type of cost or the other will likely change over time due to ongoing events, e.g., because a successful control phase will modify public opinions possibly calling for a relaxation of measures. From this perspective, $\Lambda$ regulates, given “low-level” parameters such as adherence and timeliness, the entire relationship between suppression and mitigation, particularly informing about their criticalities.

Overall, the proposed results are, to the best of our knowledge, the first to fully assess the joint role of these critical control factors. In particular, they add substantial insight to previous work^39^ that focused on the effects of prioritizing indirect cost and horizon duration; however, they relied on a simple SIR framework without public health constraints.

Clearly, in addition to the proposed strengths, the present work has limitations that might call for a number of improvements. For the “open-loop” approach, it is important to appreciate that this approach focuses on preplanned learning on control measures (i.e., preparedness) and not on their implementation during an ongoing epidemic. In the latter case, other approaches involving real-time adaptive controls (e.g., closed/feedback loop) would be required^27,34^. For the choice of a finite horizon problem, this hypothesis was adopted in the vast majority of contributions on the optimal control of COVID-19 in its early epoch^9,10,20,21,25–40^. This assumption can be justified by assuming that a vaccine will be available after some time and that social distancing ends once the vaccine is available. Notably, seminal works by economists who considered an infinite time horizon^20,21^ (motivated by the randomness of vaccine arrival time) eventually worked with finite horizons. Some works complementary to the present one investigated the sensitivity of the finite-horizon optimal solution with respect to the length of the horizon as a step toward infinite-horizon problems^39,40^.

On the transmission model side, we focused on a specific model drawn from the first COVID-19 epoch. Given the documented massive age-space heterogeneity of COVID-19 (transmission, evolution to symptoms, risk of serious consequences, etc.) including chronological age-structure and spatial heterogeneity could be important improvements^21,35^. Furthermore, in practice, timely interventions with high levels of adherence are typically difficult to achieve, because individuals demand “high perceived risks” to provide a substantial policy-enhancing behavioural response. In other words, early and effective government responses will hardly favour rapid and high adherence by individuals. Since both timeliness and adherence are control parameters strongly influenced by individual behaviour, we can consider the current model a “pre-behavioural” model deserving to be amended by including endogenous agents’ responses to both epidemic trends and their control measures. Therefore, behavioural epidemiology approaches based on coupled infection-behaviour models must be considered^25,46–50^. Moreover, the adopted model is deterministic, meaning that its predictions are invalidated when the infection incidence becomes low, as typically occurs in the earlier phases of an epidemic^51^ or nearby its end^52^, or even when considering finite public health resources^43^. All these situations require the use of a stochastic modelling approach. The inability of the standard optimal control model to handle elimination strategies (which occurs in our model at very high prioritization of direct costs) is a typical example. Further, several NPIs can be jointly optimally controlled^20,21,25,31,34^. Additionally, the concept of indirect costs of a pandemic is complex from a measurement viewpoint. Essentially, every social, economic, health or relational activity that has been penalized by the restrictions adopted to mitigate the direct health effects of the pandemic represents an “indirect cost”, building an endless list inflating the cost functional of potentially redundant information. For this reason, the current approach, borrowed from early economic efforts^20^, can be considered conservative and useful, but the issue of relevantly defining indirect costs is still quite unresolved. Recent work has attempted to include in the optimization the costs of economic losses resulting from sector-specific social distancing^37^. Finally, the present model disregarded the issue of uncertainty in the structure of the transmission model and its parameters, which is clearly one pervading all questions underlying preparedness activities. We did not consider this here because our focus was on seeking social distancing principles depending on the cost structure.

All the aforementioned elements should be the object of future work. More generally, we believe that future preparedness activities should include optimal control reasoning, which is currently dramatically underused compared to other areas of mathematical modelling, on the list of public health tools. This will require going beyond works such as the present one, with the aim of developing a catalogue of optimal results based on different combinations of interventions under different settings, pandemic scenarios and associated costs. Nonetheless, we would like to point out that the elements included here (costs prioritization-effectiveness-timeliness) must remain the building blocks of such more refined formulations.

To conclude, the previous results offered clear-cut insight into the shapes of optimal social distancing and its dependence on costs and key intervention parameters. This learning is fundamental for preparedness activities and related emergency staff. This would, in turn, call for public investment in information and awareness among the people deputed to future responses (e.g., public health officers), public policy decision makers and the general population (e.g., for training towards such events).

## Methods

### Transmission model

**Figure 6:**
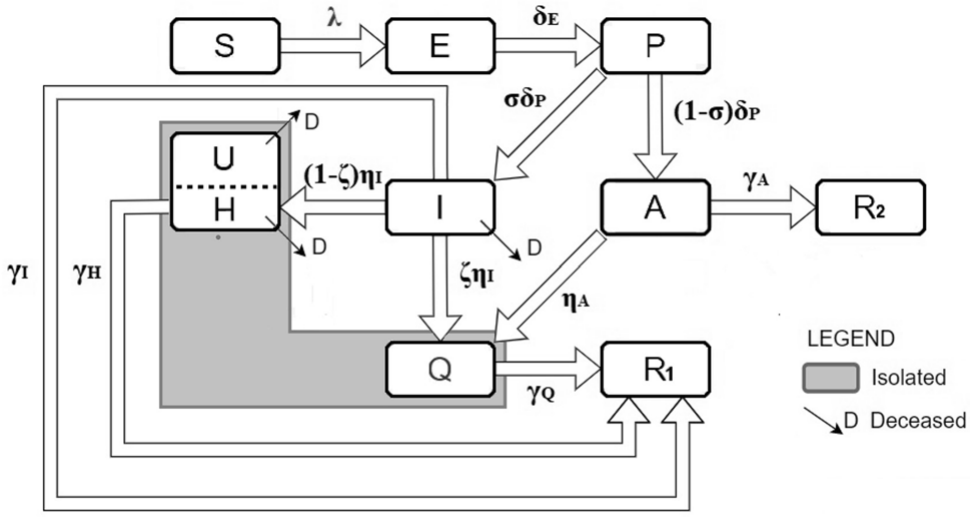
Flowchart of the adopted epidemic model and related parameters.

The adopted transmission model extends an established model for the first COVID-19 wave in Italy^41^ by including social distancing and the finiteness of hospital resources (Fig. 6). The model includes the following compartments: susceptible ($S$), exposed ($E$), presymptomatic ($P$), symptomatic infected ($I$), asymptomatic (or mild) infected ($A$), quarantined with minor symptoms ($Q$), symptomatic recovered (${R}_{1}$), asymptomatic recovered (${R}_{2}$), dead ($D$), and people requiring hospitalization ($H$). When the hospital capacity (${H}_{max}$) is reached, an untreated ($U$) class, which includes all individuals who cannot be hospitalized due to the saturation of healthcare facilities (and who die at a higher rate than those hospitalized), increases as H increases with H and H_max_. The corresponding ODEs are as follows:

2.1a $$\dot{S}=-\lambda S{\left(1-\theta L\right)}^{2}$$

2.1b $$\dot{E}=\lambda S{\left(1-\theta L\right)}^{2}-{\delta }_{E}E$$

2.1c $$\dot{P}={\delta }_{E}E-{\delta }_{P}P$$

2.1d $$\dot{I}=\sigma {\delta }_{P}P-\left({\eta }_{I}+{\gamma }_{I}+{\alpha }_{I}\right)I$$

2.1e $$\dot{A}=\left(1-\sigma \right){\delta }_{P}P-{(\gamma }_{A}+{\eta }_{A})A$$

2.1f $$\dot{H}=\left(1-\zeta \right){\eta }_{I}I-\left({\gamma }_{H}+{\alpha }_{H}\right){\text{min}}\left(H,{H}_{max}\right)-{\alpha }_{U}U$$

2.1g $$\dot{Q}=\zeta {\eta }_{I}I+{\eta }_{A}A-{\gamma }_{Q}Q$$

2.1h $$\dot{{R}_{1}}={\gamma }_{I}I+{\gamma }_{H}H+{\gamma }_{Q}Q$$

2.1i $$\dot{{R}_{2}}={\gamma }_{A}A$$

2.1j $$\dot{D}={\alpha }_{I}I+{\alpha }_{H}{\text{min}}\left(H,{H}_{max}\right)+{\alpha }_{U}U$$

with the untreated class defined by:

2.1k $$U={\text{max}}(0,H-{H}_{max})$$

The force of infection of a free epidemic ($\lambda$), which is the rate at which susceptible individuals acquire infection from infective agents (P, I, A) at their specific rates $(\beta$) in the absence of control measures, is given by:

2.2 $$\lambda =\frac{{\beta }_{P}P+{\beta }_{I}I+{\beta }_{A}A}{S+E+P+I+A+R}$$

where the denominator includes the socially active population. Deaths occur among H, U, I individuals. The model parameters are described in Table 1. The control variable $L(t)$ (Eqs. (2.1a), (2.1b)) specifies the fraction of the population targeted for isolation by social distancing, whose effects are further modulated by *adherence* $\theta (\theta \in \left(\mathrm{0,1}\right))$, i.e., the fraction of targeted individuals actually adhering to restrictions^20,21^. Given the basic reproduction number ${R}_{0}$ of model (2.1)^41^, the corresponding controlled number (CRN) is given by ${R}_{C}\left(t\right)={R}_{0}{\left(1-\theta L\left(t\right)\right)}^{2}$, while the effective reproduction number is given by ${R}_{E}\left(t\right)={R}_{C}\left(t\right)S(t)$.

**Table 1:** Parameters of the optimal control problem.

Parameter	Description	Unit	Value or range [ ]	Source
Epidemiological parameters				
$${{\text{R}}}_{0}$$	Basic reproduction number (in the absence of any control interventions)	–	3.6	Lit^41^
$${\beta }_{P}$$	Pre-symptomatic transmission rate	$$da{y}^{-1}$$	0.3983	Lit^53–55^
$${\beta }_{I}$$	Symptomatic transmission rate	$$da{y}^{-1}$$	0.6277	Lit^41,56^
$${\beta }_{A}$$	Asymptomatic transmission rate	$$da{y}^{-1}$$	0.2828	Lit^41^^.^^54,56^
$${\delta }_{E}$$	Latency rate ($$1/{\delta }_{E}$$ = mean latency period)	$$da{y}^{-1}$$	1/3.32	Lit^41,57^
$${\delta }_{P}$$	Post-latency rate	$$da{y}^{-1}$$	1/1.88	Lit^41^
$$\sigma$$	Probability to manifest symptoms	–	0.25	Lit^41,58^
$${\eta }_{I}$$	Detection rate of symptomatic people	$$da{y}^{-1}$$	1/4.05	Lit^41^
$${\eta }_{A}$$	Detection rate of asymptomatic people	$$da{y}^{-1}$$	$${\eta }_{I}/2$$	Lit^41^
$$\zeta$$	Probability of being hospitalized	–	0.40	Lit^41,58^
$${\gamma }_{A}$$	Recovery rate of asymptomatic	$$da{y}^{-1}$$	1/7	Lit^41,59^
$${\gamma }_{H}$$	Recovery rate of hospitalized people	$$da{y}^{-1}$$	1/14	Lit^41^
$${\gamma }_{I}$$	Recovery rate of symptomatic	$$da{y}^{-1}$$	1/14	Lit^41^
$${\gamma }_{Q}$$	Recovery rate of quarantined individuals	$$da{y}^{-1}$$	1/14	Lit^41^
$${\alpha }_{I}$$	death rate of symptomatic	$$da{y}^{-1}$$	1/24	Lit^41^
$${\alpha }_{H}$$	Death rate of hospitalized (H) people	$$da{y}^{-1}$$	1/24	Lit^41^
$${\alpha }_{U}$$	Death rate of untreated (U) people	$$da{y}^{-1}$$	$$\left(1+f\right){\alpha }_{H}, f>1$$	Free
$${H}_{max}$$	Maximal capacity of hospitals	$$-$$	195,000	Italian NIH^60^
Social distancing & cost parameters				
$$\theta$$	Adherence (Effectiveness) of social distancing	–	0.7 [0–1]	Free
$${L}_{max}$$	Upper bound of social distancing	–	0.7	Lit^20^
$$\Lambda$$	Prioritization to indirect costs	–	[0, 1]	Free
$${t}_{s}$$	Intervention delay	*days*	[0, 150]	Free
$$\omega$$	Average daily wage	$	32,500/365	World Bank 2020
$$1/r$$	Numbers of life years lost by those dying of COVID	$$year$$	20	Lit^20,61^
$$\tau$$	Availability of post-infection testing	–	1	Lit^20^
$${\alpha }_{1}$$	Average hospitalization cost per patient	$	2275.20	Lit^62^
$${p}_{y}$$	Fraction of under-65 in deceased population	–	0.151	Lit^60^
$$vsl$$	Value of a Statistical Life ($$365\omega /r$$)	$	650,000	Lit^20^
$$T$$	Horizon length	$$days$$	365	Fixed

Parameters borrowed from the literature are denoted as “Lit”, while free simulation parameters are specified as “Free” in the “Source” column.

### The cost functional and the optimal control problem

The optimal control problem seeks the optimal social distancing (or “lockdown policy”) action $\overline{L }\left(t\right)$ that minimizes the cost functional

2.3 $$C=\Lambda {C}_{ec}+\left(1-\Lambda \right){C}_{he}$$

Given the epidemic system (2.1)-(2.2). In particular, ${C}_{he}$ and ${C}_{ec}$ quantify the total “direct” health cost of the epidemic and its “indirect” (i.e., societal) cost, respectively. The weight $\Lambda$ $(0\le\Lambda \le 1$) reflects the prioritization attributed to indirect costs by the public policy planner.

The adopted structure of costs ${C}_{ec}$ follows influential economic approaches^20,21^. Letting $T$ denote the length of the control horizon, the indirect costs ${C}_{ec}$ reflect the corresponding loss of working days due to social distancing over the entire horizon:

2.4 $${C}_{ec}={\int }_{0}^{T}\omega \left\{L(t)\left[N\left(t\right)-\left({W}_{Q}\left(t\right)+\tau {R}_{1}\left(t\right)\right)\right]+{W}_{Q}\left(t\right)\right\}dt$$

where ${W}_{Q}\left(t\right)$ is the size of the population unable to work because of quarantine or serious illness (i.e., ${W}_{Q}\left(t\right)=Q\left(t\right)+H\left(t\right)$) and $\omega$ is the average daily wage. The term $L\left(t\right)N(t)$ represents the population subject to restriction measures. We also included the possibility of readmission of confirmed recovered individuals among those who received a recovery confirmation (${R}_{1}(t)$) owing to the availability of postinfection testing, where $\tau =1$ ($\tau =0$) denotes whether a test is available or not^20^.

The direct cost of the epidemic (${C}_{he}$)

2.5 $${C}_{he}={\int }_{0}^{T}{\alpha }_{1}H\left(t\right)dt+{p}_{y}\frac{365\omega }{r}D(T)$$

is taken as the sum of (i) the integrated number of hospitalized people ($H\left(t\right)$), representing total hospitalization time, evaluated at the (constant) average daily hospitalization cost ${\alpha }_{1}$ due to COVID-19^62,63^ and *(ii)* the overall cost due to total COVID-19 deaths throughout the entire horizon, $D(T)$. The latter formulation also follows established economic approaches^20,21^ and quantifies the cost of a death of a worker by the monetary value of a statistical life, which is computed by the product between the worker’s average yearly income ($\omega$) and her expectation of residual life ($1/r$)^61^. The constant ${p}_{y}$ denotes the fraction of the work-age population.

Finally, the overall death rate was computed as the weighted average of the underlying group-specific death rates (${\alpha }_{I},{\alpha }_{H},{\alpha }_{U}$). Details on the computations of the solution to the OC problem are reported in the SM.

### Model parametrization

Table 1 lists all the model parameters with the corresponding baseline values. In particular, the key parameters of this study, namely, cost prioritization ($\Lambda$), adherence/effectiveness ($\theta$) and intervention delay (${t}_{s}$), are taken as free parameters. The per capita GDP ($\omega$) was drawn from prepandemic Italian data. The hospitalization cost (${\alpha }_{1}$) is computed as a weighted average of hospital costs/day and ICU costs/day. The initial conditions of the state system (2.1) are set as the classical epidemic condition, with a few exposed individuals (e.g., 10) in an otherwise fully susceptible population. When intervention delays are considered, the optimal control solution is initialized after a duration ${t}_{s}$ of free epidemic growth from such initial conditions.

### Supplementary Information

Supplementary Information.

## Data Availability

Code and original figures generated for the current study are available at https://github.com/3135163/Project/tree/main/Scientific-Reports-2023-2024/.
