# Supplementary material for: Optimal social distancing in epidemic control: cost prioritization, adherence and insights into preparedness principles
Source: Sci Rep. 2024 Feb 22;14:4365. doi: 10.1038/s41598-024-54955-4 (PMC10883963; doi:10.1038/s41598-024-54955-4)
Supplement: Supplementary file 1 — Supplementary Information. [file 41598_2024_54955_MOESM1_ESM.pdf]

Optimal social distancing in epidemic control: cost prioritization, adherence and insights into preparedness principles

Giulio Pisaneschi[1], Matteo Tarani[1], Giovanni Di Donato[1], Alberto Landi[1], Marco Laurino[2], Piero Manfredi[3,*]

[1]Department of Information Engineering, University of Pisa, Pisa, Italy;
[2]National Research Council, Institute of Clinical Physiology, Pisa, Italy;
[3,*] Department of Economics and Management, University of Pisa, Pisa, Italy; piero.manfredi@unipi.it
(Corresponding Author)

Supplementary materials

A1. Computation of the optimal control and convergence

The optimal control problem was solved numerically by MATLAB function "fmincon" (MathWorks, Inc. (2022), *MATLAB: fmincon, Solver-Based Nonlinear Optimization*, version R2022b. https://www.mathworks.com/help/optim/ug/fmincon.html), supplemented by a number of preliminary checks and heuristics to deal with local minima problems. The epidemic system was solved using a fourth order fixed step Runge-Kutta method with daily step. Due to the high number of computations required to handle the free simulation parameters, the initial control action, the large number of iterations required for convergence, and the initial epidemic conditions, the problem was parallelized on the University of Pisa cluster. Extensive preliminary checks were performed to ensure that the final result of the optimization was not affected by the form of the initial control function. We report an example of such preliminary checks in Fig. A.1 showing, for selected values of Λ, and θ set to its baseline ($\theta = 0.7$), the shape of the optimal control action $\tilde{L}(t)$ resulting from 50 different initial control actions $L_0(t)$. As the figure shows, every initial action ultimately leads to the optimal outcome, validating that the optimization process consistently converges to the same solution regardless of the initial function

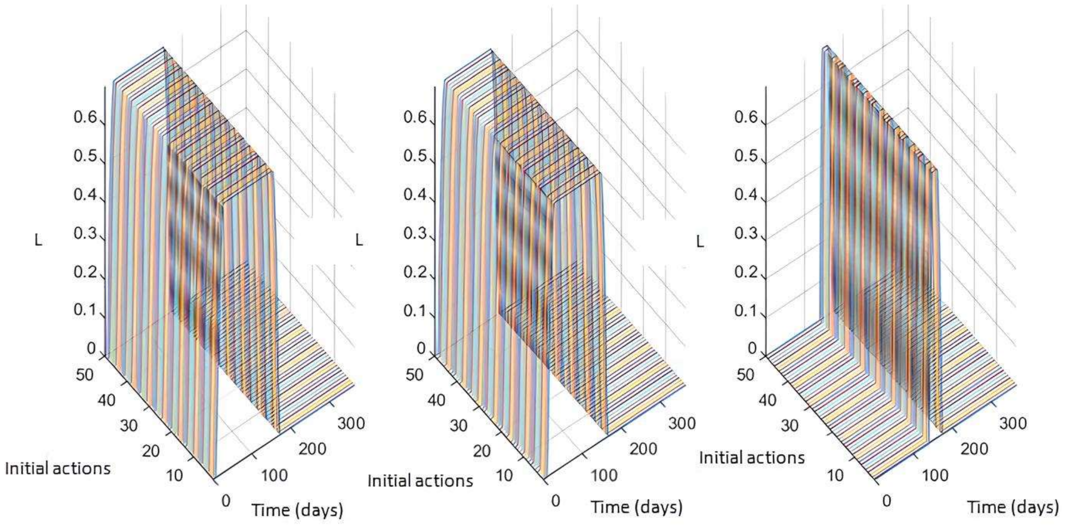

Figure A1. Optimal lockdown policies for selected values of Λ: $\Lambda = 0.06$ $(left)$; $\Lambda = 0.24$ $(centre)$; $\Lambda = 0.52$ $(right)$ confirming convergence to a unique optimal control function regardless of the form of the initial function. Initial control actions are symbolically identified by the left horizontal axis. Each value of the grid (50 values in total) on the left horizontal axis represents a different initial control function. Among such 50 initial controls, the first 11 correspond to a constant pattern while the remaining 39 correspond to a fully random pattern (generated by MATLAB function "*rand*"). This analysis allowed us to determine the appropriate number of iterations beyond which all initial functions converged to the same optimal control. Other parameters are set to their baselines (Table 1 of main text).

A2. Further results on the role of adherence

We report further results illustrating the specific effect of adherence (θ) for different levels of cost prioritization (Figs A2.1, A2.2 and A2.3). For low levels of Λ ($\Lambda = 0.12$, Fig. A2.1), high adherences (i.e., θ nearby 1 meaning almost universal acceptance of the proposed measures), the optimal distancing action (Fig. A2.1, top) yields a minimal duration of restrictions (less than four months). However, as θ decreases, the duration of optimal closures disproportionately increases to mitigate direct epidemic costs until the level $\theta = 0.32$, where adherence is so low that control measures need be maintained for almost the entire horizon. Below that threshold, adherence becomes so low to force a rapid transition from suppression to mitigation, with dramatically delayed optimal actions. The window of adherence allowing effective mitigation (preventing hospitals' overwhelming, Fig. A2.1, bottom, left) is very narrow ($0.27 < \theta < 0.32$). As adherence is further declined, mitigation becomes ineffective with a fast growth of untreated individuals and a blow-up in the mortality rate (Fig. A2.1, bottom, right).

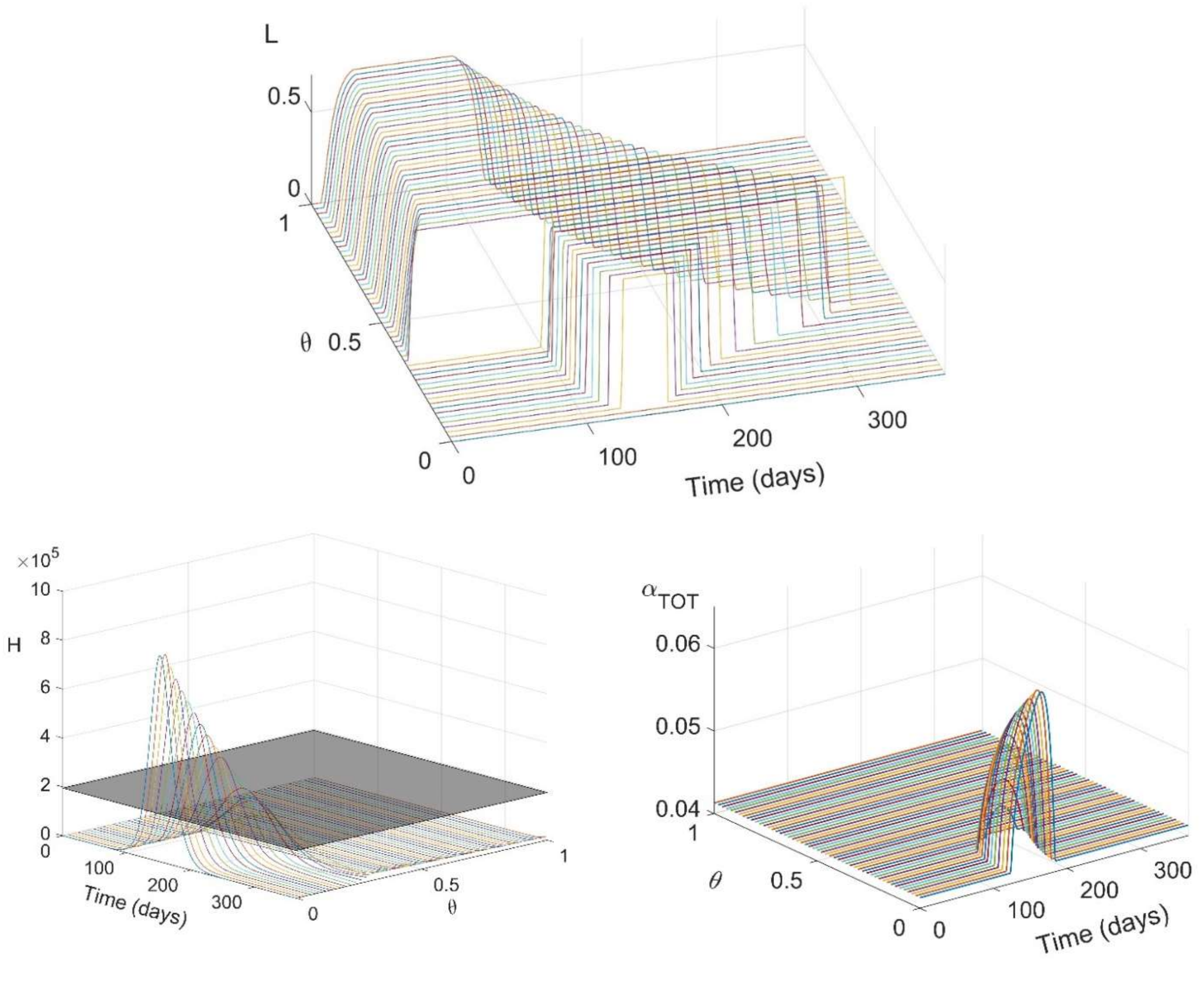

Figure A2.1. Effects of adherence to interventions (θ), for $\Lambda = 0.12$. Top: time evolution of optimal social distancing as a function of θ; centre: number of people requiring hospitalization (H) as a function of θ; bottom: mortality rate (α_{TOT}) among H people as a function of θ.

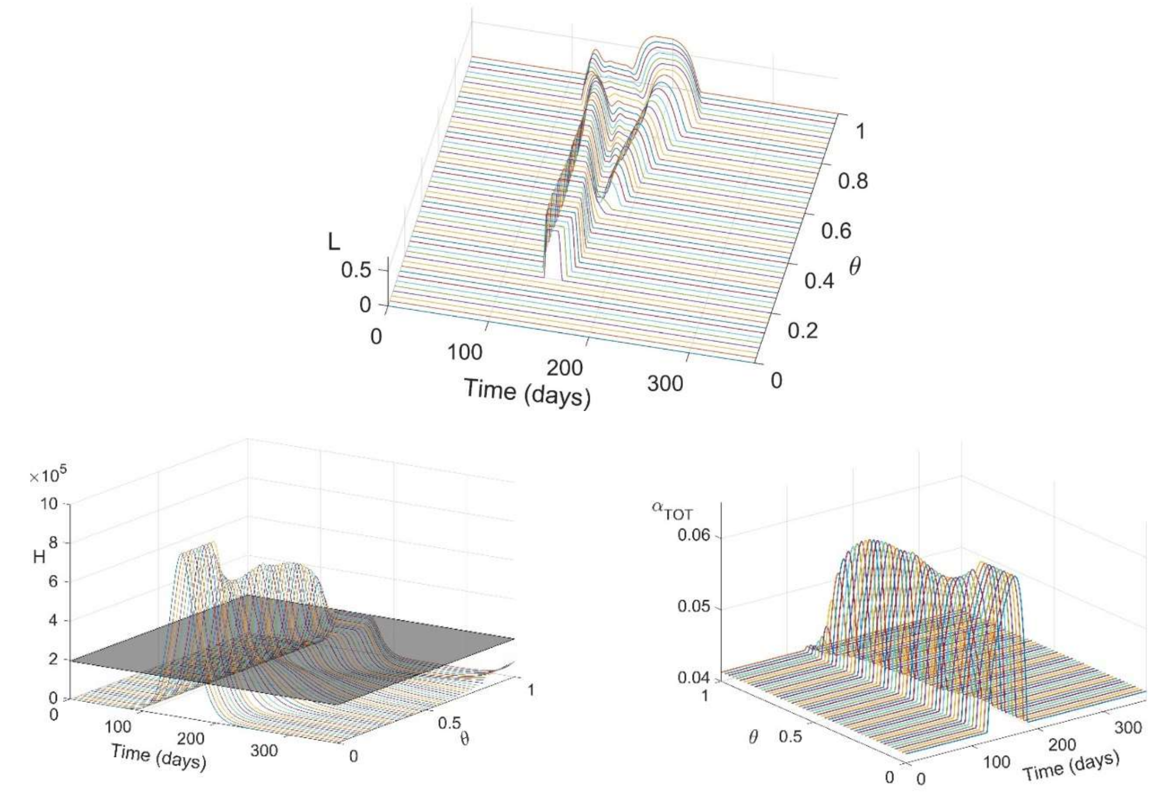

Figure A2.2. Effects of adherence to interventions (θ), for $\Lambda = 0.52$. Top: time evolution of optimal social distancing as a function of θ; centre: number of people requiring hospitalization (H) as a function of θ; bottom: mortality rate(α_{TOT}) among H people as a function of θ.

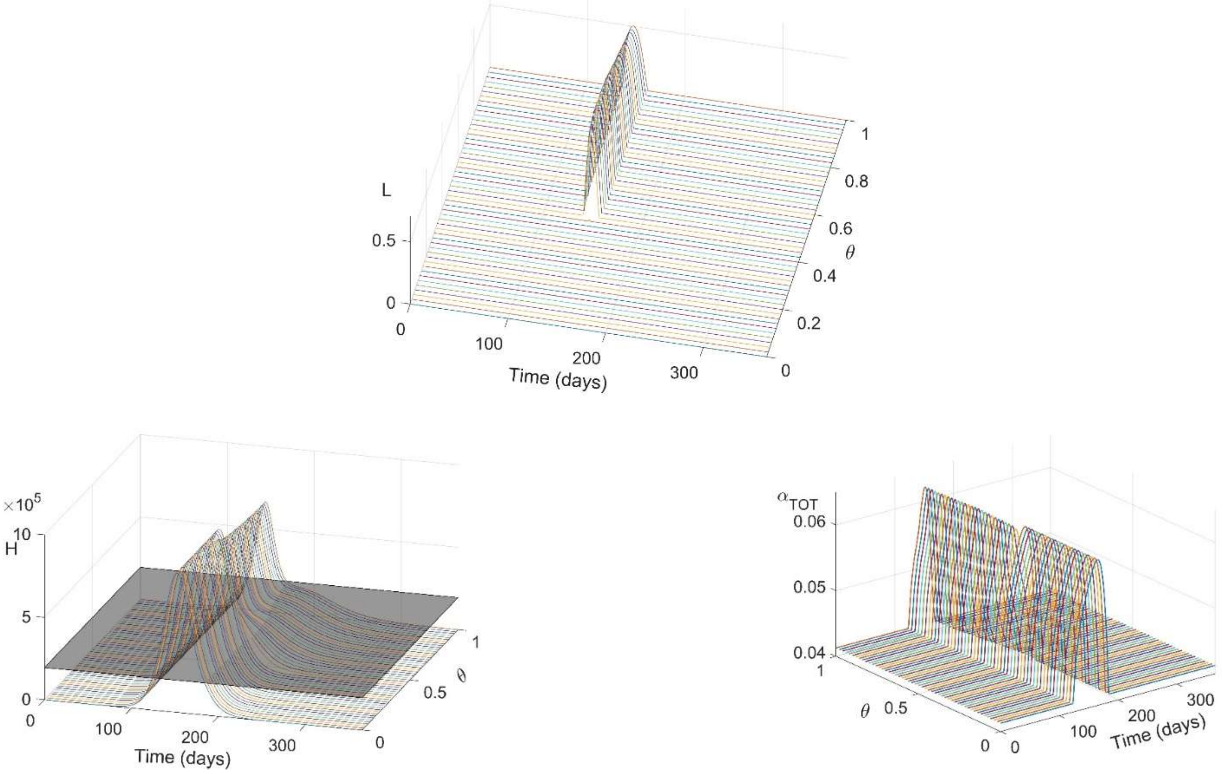

Figure A2.3. Effects of adherence to interventions (θ), for $\Lambda = 0.72$. Top: time evolution of optimal social distancing as a function of θ; centre: number of people requiring hospitalization (H) as a function of θ; bottom: mortality rate(α_{TOT}) among H people as a function of θ.

A3. Further results on timeliness

The following figures show the trends of optimal social distancing (L) and people requiring hospitalization (H) for two different values of Λ in the suppression region.

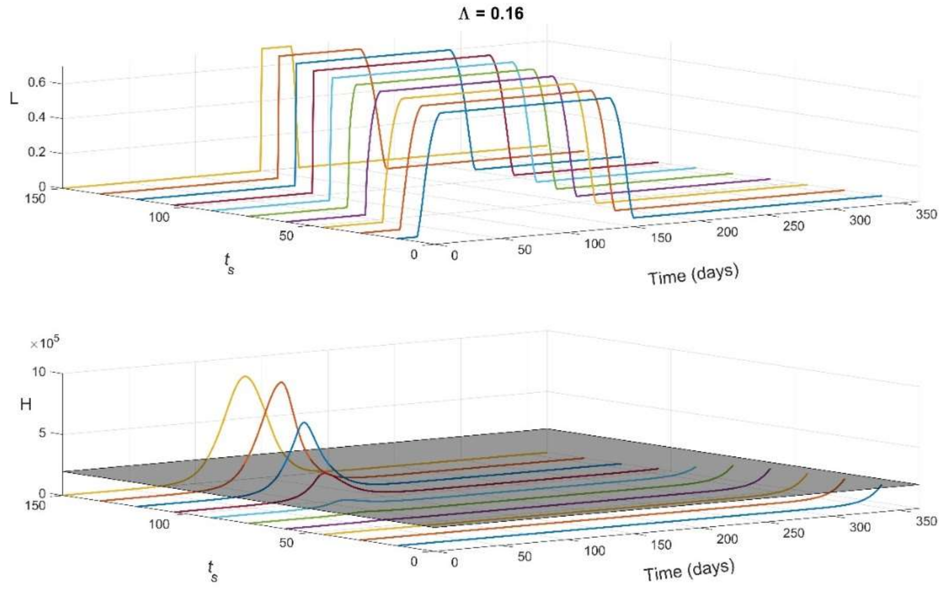

Figure A3.1. Effects of different intervention delays (t_s) under a regime of strong prioritization to direct costs ($\Lambda = 0.16$): time evolution of optimal social distancing (L(t), top) and number of people requiring hospitalization (H(t), bottom).

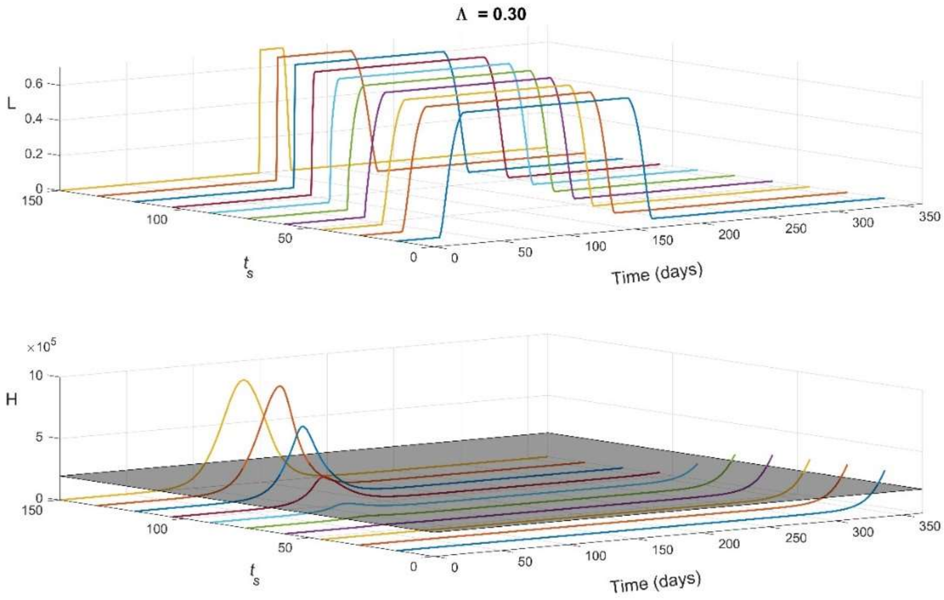

Figure A3.2. Effects of different intervention delays (t_s) under a regime of strong prioritization to direct costs ($\Lambda = 0.30$): time evolution of optimal social distancing (L(t), top) and number of people requiring hospitalization (H(t), bottom).

A.4 Additional results: relaxing the hospitals' constraint

This section reports some additional results for the special case where the finiteness of hospitals' resources is not taken into account. Though unrealistic, this case has often been considered in the optimal control analyses of the COVID-19 literature (e.g., Novak et al 2023, Aldila et al 2020). Compared to the case presented in the main text, H by definition includes all individuals requiring hospitalization without considering the U dimension. In particular, the complexity of the control action and of the various epidemiological dimensions for different levels of the relative cost of intervention is lost. As shown in Fig A.4.1 (to be compared with Fig 1 of main text): (i) as long as optimal social distancing prevents hospitalizations to overwhelm hospitals' capacity , the results are exactly the same as in the main text; (ii) however, once hospitals' saturation is approached, the pattern of the optimal action remains simple i.e., increasing Λ progressively delays optimal social distancing and reduces its intensity until when no action is predicted). Therefore, no multi-wave optimal scenarios emerge. Obviously, the razor blade effect is preserved.

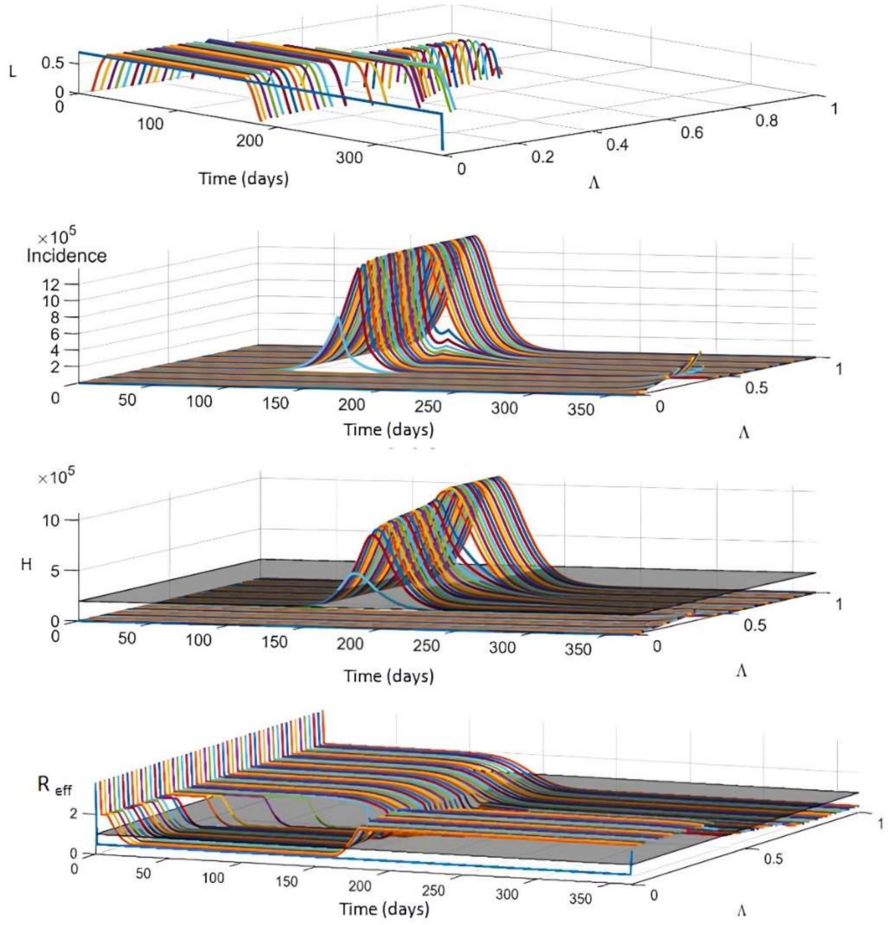

Figure A4. Time evolution of: (i) optimal social distancing (top graph), (ii) the corresponding incidence of new infections $\lambda S(1-\theta L)^2$ (second row), (iii) the number of current hospitalizations (i.e., compartment H, third row), and (iv) the effective reproduction number (bottom graph), for different values of the relative cost of interventions Λ. The shaded plane in the hospitalization graph (still reflecting the maximum hospital capacity in Italy) is shown for comparison with Fig 2 in the main text. The shaded plane in the graph of effective reproduction represents the unit threshold. Note that for $\Lambda = 0$ there is a "total" optimal control action that lasts for the entire horizon. Adherence is set to its baseline value $\theta = 0.7$. All other parameters and initial conditions are as in Fig 2 of the main text. In particular, the following critical values/windows appear: (i) $\Lambda = 0$: total control; (ii) $0 < \Lambda < 0.38$, suppression by sufficiently early/intense lockdown; (iii) $\Lambda \cong 0.38$ razor blade with rapid transition from suppression to mitigation; (iv) $0.38 < \Lambda < 0.72$ mitigation of decreasing quality; (v) $0.72 < \Lambda < 1$, 'do nothing'.
